# HIV-associated disparities in interdialytic weight gain and depressive symptomatology among male hemodialysis patients: A comparative analysis of fluid management adherence

**DOI:** 10.1097/MD.0000000000046134

**Published:** 2025-11-28

**Authors:** Fangyan Xu, Shuying Zhou, Huili Li, Huanhuan Yang, Jian Liu, Congfei Wang, Xuefen Wang, Gongzhen Cui, Xiaofeng Zheng, Zhangqing Zhou

**Affiliations:** a Department of General Medicine, Xixi Hospital of Hangzhou, Hangzhou, Zhejiang, China.

**Keywords:** chronic kidney disease, depressive symptoms, hemodialysis, HIV, interdialytic weight gain

## Abstract

Chronic kidney disease is common among people living with human immunodeficiency viruses (PLWH). Effective volume management is crucial for patients undergoing hemodialysis. This study analyzed the clinical characteristics and treatment status of male PLWH on hemodialysis, investigated differences in volume control compared to non-HIV-infected male patients, and further investigated the association between fluid management adherence and depressive symptoms in this population. This analytical study included 37 non-HIV-infected and 20 PLWH male hemodialysis patients. Baseline data and health indicators, including hematological parameters, electrolyte levels, lipid profiles, inflammatory markers, Kt/V, and urea reduction ratio values, were assessed. Interdialytic weight gain (IDWG) was measured over 1-month to determine the mean IDWG and its percentage relative to the dry body weight. These patients were then invited to complete the hospital anxiety and depression scale (HADS). There were no significant differences in erythrocyte, albumin, blood glucose, lipid, urea, creatinine, uric acid, potassium, parathyroid hormone, hypersensitive C-reactive protein, and dialysis adequacy between the 2 groups. PLWH on hemodialysis had lower blood calcium levels (2.1 ± 0.2 vs 2.2 ± 0.1, *P* < .05) and higher phosphorus levels (2.2 ± 0.7 vs 1.8 ± 0.5, *P* < .05). The mean IDWG for non-HIV-infected patients was 2.1 ± 0.8 kg, while for PLWH it was 3.1 ± 0.9 kg (*P* < .001). As a percentage of dry body weight, mean IDWG was 4.9 ± 1.5% for PLWH compared to 3.3 ± 1.1% for non-HIV-infected patients (*P* < .001). PLWH receiving hemodialysis demonstrated significantly higher HADS-D scores (6.7 ± 2.6 vs 4.2 ± 2.4, *P* < .01) and total HADS scores (10.7 ± 5.3 vs 6.8 ± 3.6, *P* < .01). Patients with IDWG exceeding 4% of dry body weight demonstrated significantly higher HADS-D scores (6.6 ± 2.5 vs 3.9 ± 2.4, *P* < .01) and total HADS scores (10.1 ± 4.0 vs 6.7 ± 4.5, *P* < .01) compared to those with lower fluid gains. PLWH undergoing hemodialysis exhibited poorer weight management and more severe depressive symptoms. Depressive symptomatology may adversely influence adherence to fluid management in patients undergoing hemodialysis.

## 1. Introduction

AIDS, which is caused by human immunodeficiency viruses (HIV), is a systemic and progressive disease that primarily targets the immune system. Without treatment, patients are at risk of severe infections and cancers, leading to death in advanced stages. As of 2022, approximately 39 million people worldwide are living with HIV, including 1.3 million new infections that year.^[1]^

Kidney injury is a significant complication of HIV infection, potentially caused by direct kidney damage from intrarenal HIV infection and gene expression, immune dysregulation, treatment-related toxicity, comorbidities, and co-infections.^[2]^ The incidence of HIV-associated nephropathy has decreased with the advent of antiretroviral therapy (ART).^[3]^ Similar to the general population, the most common causes of chronic kidney disease in people living with HIV (PLWH) are hypertension and diabetes.^[4]^ Studies have shown that PLWH are approximately 4 times more likely to develop type 2 diabetes and respond less favorably to treatment.^[5]^ According to statistics, the risk of end-stage kidney disease in PLWH is 2 to 20 times higher than that in the general population.^[4]^ As ART improves the life expectancy of PLWH, the need for dialysis is also increasing.

In dialysis patients, chronic volume overload can lead to hypertension, left ventricular hypertrophy, heart failure, pulmonary edema, and an increased risk of mortality.^[6]^ Interdialytic weight gain (IDWG) refers to the weight gain experienced by patients undergoing hemodialysis between dialysis sessions. Previous studies have indicated that IDWG should not exceed 3 kg.^[7]^ Some scholars have proposed that IDWG should be <4.0% to 4.5% of dry weight.^[8]^ High IDWG is associated with an increased risk of all-cause and cardiovascular mortality.^[8]^ Furthermore, male patients tend to exhibit higher IDWG.^[9]^ Nonadherence to treatment (diet/fluid restriction, medications, and dialysis) is prevalent among patients with end-stage kidney disease undergoing dialysis and is linked to an elevated risk of mortality and adverse health outcomes.^[10]^ Several studies have identified depression as a critical factor influencing treatment adherence in this population.^[11]^

With the growing number of PLWH receiving hemodialysis, attention to their quality of life and prognosis has increased. It is currently unclear whether IDWG differs between PLWH and non-HIV-infected patients. This study aimed to analyze the characteristics and treatment status of male PLWH on hemodialysis and investigate the differences in volume control compared to non-HIV-infected male patients. We further explored whether weight gain is associated with depression. This study seeks to assist clinicians in making timely interventions, improving patient outcomes, and reducing financial burdens.

## 2. Materials and methods

This study reviewed the medical records of 57 male patients who underwent hemodialysis at our center between September, 2023 and December, 2024. The cohort comprised 37 non-HIV-infected individuals and 20 PLWH. The inclusion criteria were: age 18 to 85 years; undergoing regular hemodialysis for ≥3 months. Exclusion criteria were: history of acute coronary syndrome, stroke, or major surgery within the past month; previous malignant tumors or severe mental illness; evident infections or fever; hepatitis *B* or syphilis; and interruption of hemodialysis or ART during the treatment period.

Baseline data including age, blood pressure, and dialysis duration were collected. Fasting venous blood samples were collected to measure the red blood cell count, hemoglobin, ferritin, parathyroid hormone, phosphorus, calcium, potassium, albumin, glucose, uric acid, total cholesterol, triglycerides, and hypersensitive C-reactive protein. Venous blood was also collected before and after hemodialysis sessions to calculate the Kt/V and urea reduction ratio based on creatinine and urea levels. Adequate dialysis was defined as Kt/V ≥ 1.2 and urea reduction ratio ≥ 65%.

IDWG was defined as the difference between the pre- and post-dialysis weights from the previous session. IDWG data were collected over a month and the mean IDWG was calculated. The mean IDWG as a percentage of dry body weight was determined and analyzed. An IDWG >4.0% of dry body weight is considered excessive IDWG.^[12]^

These patients were then invited to complete the hospital anxiety and depression scale (HADS), HADS-A, and HADS-D subscales. Items were scored on a 4-point scale with total scores per subscale ranging from 0 to 21. A recommended screening cutoff score of ≥8 was used as an indication of anxiety or depression.^[13]^

All the clinical studies were conducted in accordance with the principles of the Declaration of Helsinki. This study was approved by the Ethics Committee of Hangzhou Xixi Hospital (2024-089).

Continuous variables were analyzed using the *t* test or Mann–Whitney *U* test based on data distribution, while categorical variables were compared using the chi-square test to evaluate baseline characteristics. Normally distributed continuous variables are presented as mean ± standard deviation and skew-distributed variables are presented as interquartile ranges. Categorical variables were expressed as percentages. All analyses were conducted using IBM SPSS Statistics 25 (IBM Corp., Armonk), with *P* < .05 considered statistically significant.

## 3. Results

**Figure 1.**
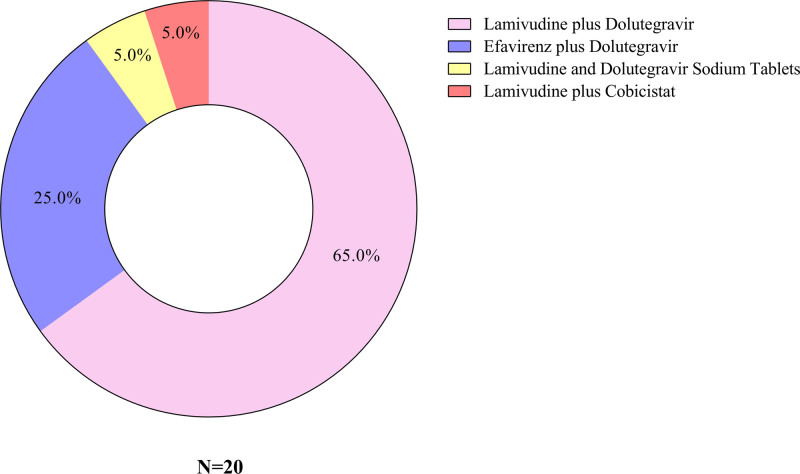
The ART for PLWH. ART = antiretroviral therapy, PLWH = people living with human immunodeficiency viruses.

None of the patients experienced serious adverse events during the study period. The clinical characteristics are summarized in Table 1. Compared to non-HIV-infected patients, PLWH were younger and had higher systolic blood pressure, whereas diastolic blood pressure did not differ significantly. Among the 20 PLWH, 30% had a history of peritoneal dialysis, which was significantly higher than that in non-HIV-infected patients. There was no significant difference in dialysis duration between the groups, and the primary dialysis access was an autogenous arteriovenous fistula. No significant differences were found in the prevalence of hypertension, diabetes, or chronic kidney disease-mineral and bone disorders. As shown in Figure 1, ART options for PLWH undergoing hemodialysis are limited due to impaired renal function. The most common regimen was lamivudine plus Dolutegravir (65.0%), followed by efavirenz plus Dolutegravir (25.0%). Other regimens included Lamivudine and Dolutegravir Sodium Tablets (5.0%), and lamivudine plus cobicistat (5.0%).

**Table 1:** The clinical characteristics of all participating groups.

		Non-HIV-infected hemodialysis patients(n = 37)	PLWH undergoing hemodialysis (n = 20)	*P*
Demographics				
Male/female		37/0	20/0	–
Age (yr)		60.7 ± 14.3	52.4 ± 15.6	.047
SBP (mm Hg)		143.0 ± 19.4	157.4 ± 32.6	.041
DBP (mm Hg)		83.0 ± 19.6	87.7 ± 11.3	.329
Dialysis mode n (%)				
Dialysis duration (mo)		33.0(9.5–49.5)	20.5(12.5–36.3)	.519
History of peritoneal dialysis		0(0%)	6(30.0%)	.001
Dialysis access	Tunneled cuffed catheter	7(18.9%)	3(15.0%)	–
	Arteriovenous fistula	29(78.4%)	17(85.0%)	–
	Arteriovenous graft	1(2.7%)	0(0%)	–
Complication, n (%)				
Diabetes		15(40.5%)	8(40.0%)	1.000
Hypertension		27(73.0%)	14(70.0%)	1.000
Medication for CKD-MBD, n (%)				
Calcitriol		21(56.8%)	14(70.0%)	.245
Phosphate binder		21(56.8%)	12(60.0%)	.519
Cinacalcet		8(21.6%)	4(20.0%)	.585
HIV information n (%)				
HIV duration (yr)		/	8.0(5.0–11.0)	–

CKD-MBD = chronic kidney disease -mineral and bone disorder, DBP = diastolic pressure, PLWH = people living with HIV, SBP = systolic pressure.

In the laboratory tests (Table 2), no significant differences were found between the 2 groups in hemoglobin, erythrocyte count, albumin, blood glucose, lipid profiles, urea, creatinine, uric acid, potassium, parathyroid hormone, hypersensitive C-reactive protein, or dialysis adequacy. However, non-HIV-infected patients had lower ferritin levels than HIV-infected patients. Despite no significant differences in medication use or bone mineral disorders, PLWH on hemodialysis had lower blood calcium (2.1 ± 0.2 vs 2.2 ± 0.1, *P* < .05) and higher blood phosphorus (2.2 ± 0.7 vs 1.8 ± 0.5, *P* < .05).

**Table 2:** Laboratory tests.

	Non-HIV-infected hemodialysis patients (n = 37)	PLWH undergoing hemodialysis (n = 20)	*P*
Hemoglobin (g/L)	114.0 ± 10.3	111.5 ± 10.4	.384
Erythrocyte (×10^9^/L)	3.9 ± 0.5	3.6 ± 0.5	.073
Ferritin (μg/L)	29.2(20.2–65.6)	70.1(42.0–189.4)	.005
Albumin (g/L)	37.9 ± 3.4	36.8 ± 3.6	.274
Glucose (mmol/L)	8.3(6.1–10.4)	7.5(5.4–9.4)	.345
Total cholesterol (mmol/L)	3.4 ± 0.8	3.2 ± 0.9	.473
Triglycerides (mmol/L)	2.0 ± 1.7	2.0 ± 1.1	.901
Creatinine (μmol/L)	378.0(324.0–751.0)	412.0(298.0–841.0)	.874
Urea (mmol/L)	8.8(6.55–17.9)	9.0(7.6–21.5)	.477
Uric acid (μmol/L)	122.7(98.15–356.3)	132.2(108.1–418.4)	.385
Potassium (mmol/L)	3.6(3.3–4.7)	3.5(3.3–4.3)	.564
Calcium (mmol/L)	2.2 ± 0.1	2.1 ± 0.2	.001
Phosphorus (mmol/L)	1.8 ± 0.5	2.2 ± 0.7	.022
PTH (pg/mL)	215.0(149.4–380.2)	282.7(148.2–328.1)	.668
hs-CRP (mg/L)	3.1(1.8–7.0)	4.5(2.3–6.9)	.353
Kt/V	1.4 ± 0.2	1.3 ± 0.2	.367
URR	68.9 ± 4.1	66.3 ± 4.6	.104

hs-CRP = hypersensitive C-reactive protein, PLWH = people living with HIV, PTH = parathyroid hormone, URR = urea reduction ratio.

**Figure 2.**
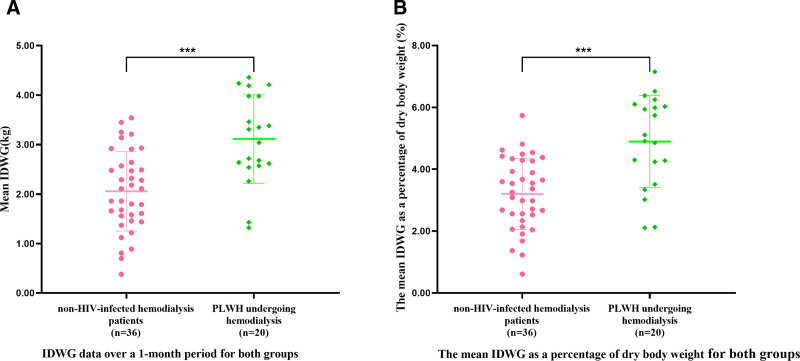
IDWG between the 2 groups. (A) IDWG data over a 1-month period for both groups. (B) The mean IDWG as a percentage of dry weight for both groups. IDWG = interdialytic weight gain.

After excluding 1 patient with a 24-hour urine volume >1500 mL who did not require dehydration during hemodialysis, we compared the IDWG between the 2 groups. The mean IDWG was 2.1 ± 0.8 kg for non-HIV-infected patients and 3.1 ± 0.9 kg for PLWH on hemodialysis, showing a significant difference (*P* < .001; Fig. 2A). Figure 2B shows the mean IDWG as a percentage of dry body weight. PLWH on hemodialysis had poorer weight management and higher water intake, resulting in a mean IDWG of 4.9 ± 1.5% of dry body weight compared to 3.3 ± 1.1% for non-HIV-infected patients. (*P* < .001).

**Figure 3.**
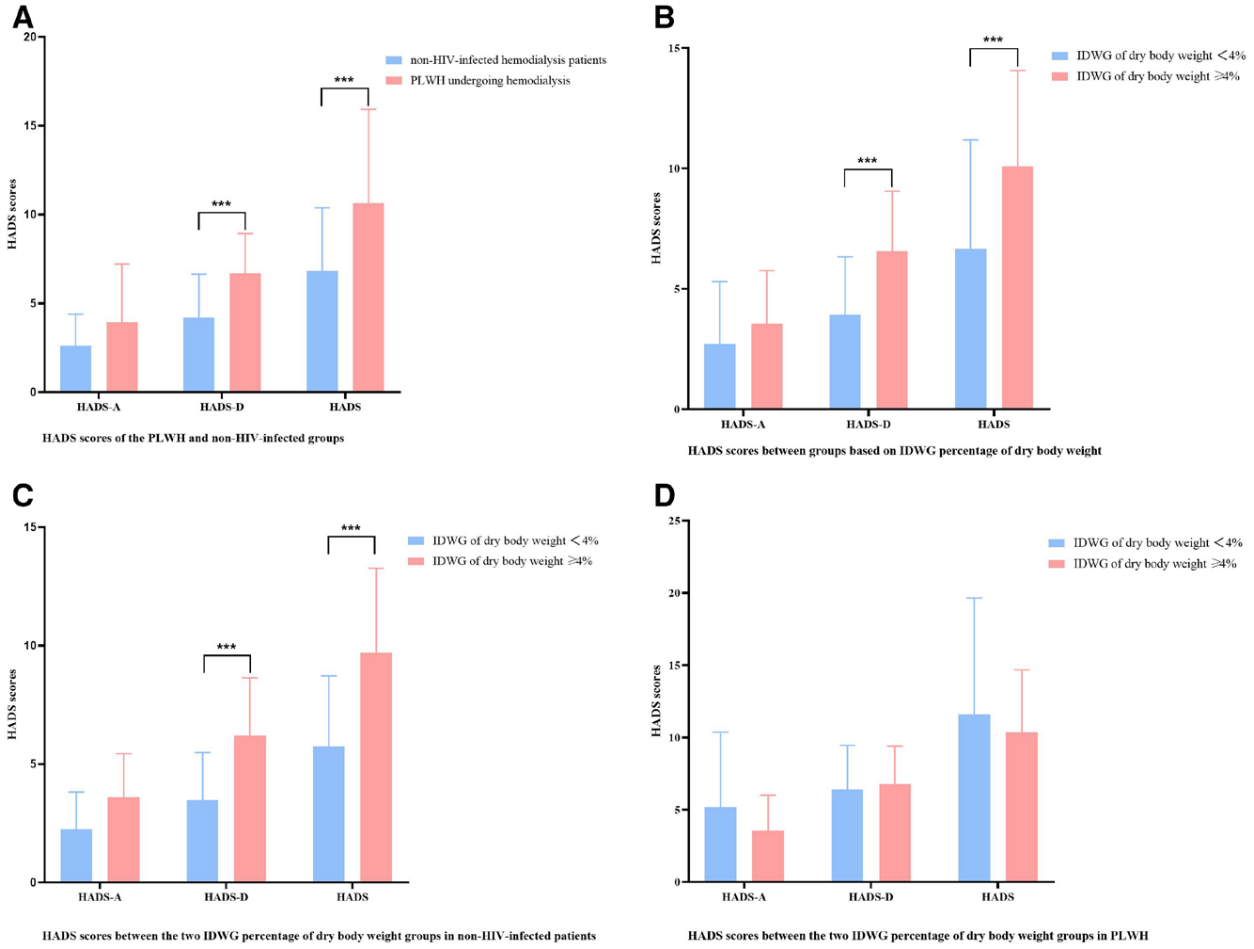
The HADS scores between the 2 groups. (A) HADS scores of the PLWH and the non-HIV–infected groups. (B) HADS scores between groups based on IDWG percentage of dry body weight. (C) HADS scores between the 2 IDWG percentage of dry body weight groups in non-HIV–infected patients. (D) HADS scores between the 2 IDWG percentage of dry body weight groups in PLWH. HADS = Hospital Anxiety and Depression Scale, HIV = human immunodeficiency virus, PLWH = people living with human immunodeficiency virus.

Furthermore, although no significant difference was observed in HADS-A scores between the 2 groups, PLWH receiving hemodialysis exhibited significantly higher HADS-D scores (6.7 ± 2.6 vs 4.2 ± 2.4, *P* < .01) and total HADS scores (10.7 ± 5.3 vs 6.8 ± 3.6, *P* < .01; Fig. 3A). Among all patients, no significant differences in anxiety symptoms were noted across IDWG categories (Fig. 3B). However, patients with IDWG exceeding 4% of dry body weight showed significantly higher HADS-D scores (6.6 ± 2.5 vs 3.9 ± 2.4, *P* < .01) and total HADS scores (10.1 ± 4.0 vs 6.7 ± 4.5, *P* < .01) compared to those with lower fluid gains (Fig. 3B). In the non-HIV-infected group, similar results were obtained (HADS-D: 6.2 ± 2.4 vs 3.5 ± 2.0; total HADS: 9.7 ± 3.6 vs 5.7 ± 3.0, *P* < .01, Fig. 3C). However, among PLWH, patients with IDWG exceeding 4% of dry body weight did not exhibit significantly higher HADS-A, HADS-D, or total HADS scores than those with lower fluid retention (Fig. 3D).

## 4. Discussion

Cardiovascular morbidity and mortality are significantly higher in patients undergoing maintenance hemodialysis, with an adjusted risk of death approximately tenfold that of the general population.^[14]^ Studies show that cardiovascular events such as sudden death and acute heart failure in hemodialysis patients follow a distinct temporal pattern, with incidences on Mondays or Tuesday being 25% to 40% higher than on other weekdays.^[14]^ This is likely due to the longer interval between the last dialysis session and Monday or Tuesday for patients on a 3-times-per-week schedule, leading to increased fluid overload. Fluid accumulation gradually increased during the interdialytic period and was removed during the dialysis sessions, causing periodic cardiovascular stress. Excessive fluid accumulation exacerbates this stress.^[14]^ Younger patients, those with higher education levels, and those with longer dialysis durations tend to have better weight management knowledge, whereas elderly patients show poorer fluid management control.^[15]^ In this study, we found that despite being younger, PLWH on hemodialysis had a significantly higher IDWG (3.1 ± 0.9 kg) than non-HIV-infected patients. These findings are inconsistent with those of the previous studies. According to data from the United States renal data system, a high IDWG (>4.8%) is associated with increased mortality.^[9]^ In our study, the mean IDWG was 4.9 ± 1.5% of the dry body weight. PLWH on hemodialysis had significantly higher blood phosphorus levels, which were closely linked to dietary intake, similar to IDWG. Ensuring high treatment adherence is crucial for reducing complications and enhancing hemodialysis efficacy. Poor adherence can lead to weight gain, hyperphosphatemia, hyperkalemia, and decreased serum albumin level.^[16]^ Multiple studies have confirmed that the implementation of psychoeducational interventions significantly enhances adherence to prescribed dietary and fluid restrictions, as quantified by standardized compliance metrics.^[17]^ Depression has emerged as a critical factor that influences adherence. More than 2-thirds of dialysis patients exhibit depressive symptoms, with those on dialysis for <1 year showing a higher prevalence than those on dialysis for 2 years or longer.^[18]^ Approximately 15% to 20% of hemodialysis patients experience anxiety, depression, and other forms of psychological distress, which may contribute to an increased risk of mortality.^[19]^ This phenomenon is linked to factors such as restricted physical activity owing to dialysis requirements, stringent dietary limitations, economic pressures, partial loss of work capacity, and social disruptions. Our findings confirmed that patients with IDWG exceeding 4% of dry weight demonstrated significantly elevated depression scores, a pattern consistent with previous reports in the hemodialysis population. Our findings corroborate that patients with IDWG > 4% of dry weight exhibit significantly higher depression scores, a pattern consistent with prior reports in the hemodialysis population. Although no significant differences in HADS scores were observed across IDWG categories in PLWH, this null result may be attributable to universally elevated baseline depressive symptoms within this population, compounded by the limited statistical power inherent in our cohort size.

Although ART has transformed HIV into a manageable chronic disease, it does not cure HIV. Patients face both physical pain from the disease and societal stigma, leading to psychological burdens, such as shame, worthlessness, pessimism, and depression. These issues significantly impair treatment adherence and quality of life.^[20,21]^ The prevalence of depression in PLWH is 2 to 4 times higher than that in the general population.^[22]^ Our study provides the first evidence that PLWH undergoing hemodialysis demonstrated significantly higher HADS-D and total HADS scores compared to their non-HIV-infected counterparts, indicating more severe depressive symptomatology in this population. These clinical observations align with our routine clinical assessment of psychological distress patterns in this patient cohort. For this subset of patients, the medical staff provided weekly education on dietary structure and fluid intake. However, many patients were either unwilling or only minimally cooperative with treatment, possibly because of negative emotional states. Many expressed sentiments such as “life are too difficult” and advocated a “carpe diem” attitude, believing that strict adherence would not improve quality of life.

Among the 20 PLWH receiving hemodialysis at our center, 3 initially chose peritoneal dialysis but transitioned to hemodialysis due to recurrent abdominal infections and inadequate dialysis efficacy. Compared with hemodialysis, peritoneal dialysis is easier to implement, requires fewer healthcare visits, incurs lower out-of-pocket costs, and results in less productivity loss.^[23]^ These factors make it particularly suitable for remote areas or regions with limited HIV-specific dialysis resources.^[24]^ Because dedicated hemodialysis machines for PLWH are not available in all regions, some PLWH cannot disclose to their families or friends why they choose distant dialysis centers. This may explain the higher preference for peritoneal dialysis among PLWH in our study compared to non-HIV-infected patients. Hemodialysis is both physically and psychologically challenging in PLWH. This cascade could explain, at least in part, that there was a greater tendency for depression in our PLWH receiving hemodialysis than in the non-HIV-infected controls.

Our study identified several previously overlooked phenomena, and raised important considerations relevant to our research. Nonetheless, certain limitations persist in this study. The small sample size may introduce bias, and there is limited data on PLWH women receiving hemodialysis. Additionally, 3 methodological constraints warrant consideration: The HADS, while validated in dialysis populations, remains susceptible to self-report biases, particularly in cognitively impaired patients; lack of multimodal verification using structured clinical interviews (e.g., MINI) or neuroendocrine markers (e.g., cortisol/DHEA ratios) precludes comprehensive depression characterization; and single-timepoint assessment cannot distinguish transient mood fluctuations from chronic depressive states. Therefore, large-scale, multicenter studies are needed to further validate the relationship between IDWG and depression.

In summary, compared to non-HIV-infected hemodialysis patients, PLWH men receiving hemodialysis had a higher IDWG and a greater mean IDWG as a percentage of dry body weight, along with more severe depressive symptoms. Notably, patients with elevated IDWG as a percentage of dry body weight showed significantly higher depression scores, suggesting that depressive symptomatology may adversely influence fluid management adherence in hemodialysis patients.

## Acknowledgments

We want to thank all participants in this study.

## Author contributions

**Conceptualization:** Fangyan Xu.

**Data curation:** Fangyan Xu, Shuying Zhou.

**Formal analysis:** Huanhuan Yang, Jian Liu, Congfei Wang, Xiaofeng Zheng.

**Methodology:** Fangyan Xu, Shuying Zhou, Huili Li, Xuefen Wang, Gongzhen Cui.

**Supervision:** Zhangqing Zhou.

**Validation:** Zhangqing Zhou.

**Writing – original draft:** Fangyan Xu.

**Writing – review & editing:** Zhangqing Zhou.
